# Response of Nitrogen and Potassium Fertigation to “Waris” Almond (*Prunus dulcis*) under Northwestern Himalayan Region of India

**DOI:** 10.1155/2014/141328

**Published:** 2014-01-22

**Authors:** Dinesh Kumar, N. Ahmed

**Affiliations:** Central Institute of Temperate Horticulture, Old Air Field, Rangreth, Srinagar, Jammu and Kashmir 190007, India

## Abstract

A field experiment was conducted on almond (*Prunus dulcis*) to study the effect of N&K fertigation on growth, yields and leaf nutrient status over two seasons (2011 and 2012) in Srinagar, Jammu and Kashmir, India. There were six treatments, namely, T_1_—100% recommended dose of fertilizers as soil application, T_2_—100% RDF through fertigations, T_3_—75% RDF through fertigation, T_4_—75% RDF through fertigation (split application), T_5_—50% RDF through fertigation and T_6_—50% RDF through fertigation (split application) with three replications under randomized block design. The results indicated that the maximum tree height (3.21 m and 3.56 m), nut weight (2.73 g and 1.94 g), nut yield (2.41 kg/tree and 5.98 kg/tree; 2.67 t/ha and 6.64 t/ha), and leaf nutrient content (2.34 and 2.38% N; 0.14 and 0.17% P; 1.37 and 1.41% K) were recorded in T_4_ treatment, whereas the highest TCSA of main trunk, primary, secondary, and tertiary branches (72.67 and 90.28 cm^2^; 16.75 and 24.26 cm^2^; 3.83 and 7.49 cm^2^; 0.47 and 1.23 cm^2^), canopy volume (7.15 and 8.11 m^3^), and fruit number (990 and 3083/tree) were recorded in T_2_ in almond variety Waris.

## 1. Introduction

Almond (*Prunus dulcis*) that belongs to the family Rosaceae is one of the important nut crops of temperate region of India, mainly grown in Kashmir valley. In India, it is grown over an area of 23,200 hectares with an annual production of 16,300 tonnes and productivity of 0.7 t/ha as compared to other almond producing countries like Jordan (7.73 t/ha), Lebanon (5.16 t/ha), Afghanistan (4.99 t/ha), USA (4.85 t/ha), Turkey (3.23 t/ha), China (2.92 t/ha), Chile (2.89 t/ha), Uzbekistan (2.86 t/ha), Israel (2.75 t/ha), and world average (1.62 t/ha), respectively (FAO, 2010). Almond kernels are concentrated sources of energy with a significant share of fat, protein, and fibre. Fats are primarily nonsaturated, mostly oleinic and linoleic fatty acids. Nonsaturated fatty acid is important in maintaining low cholesterol levels in the blood and significant amount of micronutrients [1]. Commercial almond production in India is low considering the demand and economical potential.

Irrigation and fertilizers are the most important inputs which directly affect the plant growth, fruit yield, and quality of production. Application of fertilizers through drip irrigation is the most effective way for supplying nutrients to the plant and increases fertilizer-use efficiency. In general, most of the farmers apply the fertilizers in single soil application during dormant season and no fertilizer is applied during vegetative, flowering, and fruit growth stages, thus the effectiveness of the applied fertilizers is reduced considerably. Drip irrigation plays a major role in productivity enhancement in almond [2]. Reddy et al. [3] recorded significantly higher yield, fruit size, weight, and fertilizer-use efficiency in banana with fertigation compared to soil application in banana. Application of fertilizers through fertigation improves yield and quality in different fruit crops as reported by Chauhan and Chandel [4] in kiwifruit, Ahmad et al. [5] in cherry, Raina et al. [6] in apricot, Rao and Subramanyam [7] in pomegranate, Kumar and Pandey [8] in banana, and Singh et al. [9] in apple.

Under drip irrigation, only a portion of soil volume around each plant is wetted and thus traditional methods of fertilizers application are less effective. The limited root zone and reduced amount of mineralization in restricted wetted zone are the main reason for the reduced nutrient availability to the plants [10]. One of the major advantages of fertigation is that it permits timely application of nutrients directly to root zone, reduces leaching losses, and increases the fertilizers use efficiency [11]. The nutrient requirment of almond crop through fertigation as per the crop growth stage for better crop production. The systematic information is not available in almond especially water and nutrient management. Therefore, the present investigation was aimed to increase production and potential of almond by nitrogen and potassium fertigation in northwestern Himalayan region of India.

## 2. Materials and Methods

The experiment was conducted at the research farm of the Central Institute of Temperate Horticulture (ICAR), Srinagar, Jammu and Kashmir, India, during 2011 and 2012. The research farm at Srinagar is situated in a latitude of 34° 05′ N and longitude of 74° 50′ E and at an altitude of 1640 m above MSL. The soils of this experimental field are silty loam (39.60% sand, 24.0% silt, and 36.40% clay) with medium-to-low soil fertility status. The experimental farm falls under temperate region having cold conditions from November to February and two-year mean maximum and minimum temperature of Srinagar climate indicated that the maximum is 30°C in August and the minimum is −2.1°C in December. The average annual precipitation was 620 mm distributed erratically throughout the year during the course of investigation.

The grafted almond plants were planted in prefilled pits of 90 cm × 90 cm × 90 cm size during November, 2002, at a spacing of 3 m × 3 m in an experimental field. The recommended dose of fertilizers (RDF) was 330 gN : 45 gP : 455 gK/tree/year at the age of the 8th year. The full quantity of phosphorus in plant basin has been applied 15 days before flowering in almond. The nitrogen and potassium doses were applied through fertigation as per treatment. There were six treatments, namely, T_1_—100% recommended Dose of Fertilizers (Soil application), T_2_—100% RDF through fertigation, T_3_—75% RDF through fertigation, T_4_—75% RDF through fertigation (split application of N : K in the ratio of 2/3N : 1/3K at nut set to nut development and 1/3N : 2/3K at kernel filling to maturation stage), T_5_—50% RDF through fertigation, and T_6_—50% RDF through fertigation (split application of N : K in the ratio of 2/3N : 1/3K at nut set to nut development and 1/3N : 2/3K at kernel filling to maturation stage). The experiment was conducted under randomized block design with four replications and two plants were taken in each replication.

Water soluble fertilizers like urea as a source of nitrogen and muriate of potash as potassium were injected through drip irrigation system at weekly intervals as per crop nutrient requirement in almond. The concentration of nutrient solution passing through irrigation water was around 1.0–1.5%. A separate laterals line (16 mm) was laid for each treatment and four emitters of 4 lph (Litre per hour) capacity with pressure compensated connected with 12 mm lateral were placed equidistance in east-west north-south direction at 50% distance of canopy radius. The diameter of lateral pipe was 16 mm connected with submain pipe. The irrigation was applied throughout the growing season (till initiation of leaf fall) based on pan evaporation (80%) with the following formula:

(1) WR=[DE×CF×AA×PC]IE,

where WR is water requirement of crop under drip irrigation (litre/plant/day); DE is daily pan evaporation from class-A pan (mm); CF is crop factor; AA is area allotted to each plant (m^2^); PC is percentage of canopy (leaf coverage in relation to area allowed to plant); and IE is irrigation efficiency (0.9). The other cultural practices including weed, pest, and diseases management were followed uniformly as per recommended package of practices.

The observations on canopy volume (CV) were estimated for each individual tree using a geometrical model referred to as the “contour method” CV = [((1/4)*πabh*)/(*m*(*x*) + *m*(*y*) + 1)]. The dimensions *a* and *b* were measured of the width of the tree at the base of the canopy, perpendicular and parallel to the tree row orientation, respectively. The height of the canopy (*h*) was measured from the lowest branch to the apex. The functions *m*(*x*) and *m*(*y*) were derived to accommodate the contour of the tree [12]. CV measurements were made after harvest in October 2011 and 2012. Tree trunk girth was recorded before the execution and at the end of experiment during both years of study. A ring was made with red paint at a height of 15 cm above the ground level in each selected tree to record the trunk girth from the same point each year. The trunk cross-sectional area (TCSA) of tree was calculated by using formula TCSA = Girth^2^/4*π*. Fruit was harvested at maturity, hulled, and dried and nut weight in gram and yield per tree was recorded in kilogram.

Leaf samples were collected for leaf nutrient analysis as per procedure outlined by Chapman [13]. For macro nutrient except *N* estimation, well-ground leaf tissue was digested in di-acid mixture containing HNO_3_ and HCIO_4_ in 9 : 4 ratio for *P*, *K* by using ammonium molybdate: ammonium metavanadate using double beam UV-Vis spectrophotometer (ECIL India) and the potassium was determined by using flame photometer [14]. For leaf *N* estimation, a known weight of samples was digested with H_2_SO_4_ using 10 : 1 K_2_SO_4_ and CuSO_4_ as digestion mixture and digested at 390°C until clear digestion was obtained. Digested samples were subjected to distillation with 40% NaOH and liberated ammonia was collected H_3_BO_3_ using mixed indicator. Finally liberated ammonia was titrated against 0.1 N H_2_SO_4_ and *N* content in the leaves was expressed in percentage. The data were analyzed statistically as per Steel and Torrie [15] for interpretation of results and drawing conclusions.

## 3. Results and Discussion

### 3.1. Plant Growth Parameters

The two-year data on growth parameters showed that the plant height, canopy volume, and annual shoot extension growth differed in various fertigation treatments (Table 1). A perusal of data presented in Table 1 revealed that the application of fertilizers showed significant variations in vegetative growth of almond. Maximum plant height (3.21 m and 3.56 m) was recorded in T_4_ treatment closely followed by T_2_, T_6_, and T_1_ and significantly superior to T_3_ and T_5_ treatments, whereas maximum canopy volume and shoot extension growth were recorded with the application of recommended dose of fertilizers through fertigation and increased by 14.95% and 23.74% in canopy volume and by 17.06 and 13.84% in annual shoot extension growth over T_1_ (check). The maximum plant height that was recorded in T_4_ treatment might be due to split application of fertilizers through fertigation that enhanced fertilizer-use efficiency and saving of 33% fertilizers and the minimum that was in T_5_ treatment might be due to the insufficient application of fertilizers through fertigation (50% RDF). Shirgure et al. [16] reported less plant height and canopy volume with lower fertilizer dose in citrus. The higher uptake and accumulation of nutrients in the leaf tissue of vine fertigated with recommended dose and 3/4 of RDF might have increased the rate of various physiological and metabolic processes in the plant system, thus resulted in better growth of vines. These results are in accordance with the findings of Shirgure et al. [17] in acid lime, Chauhan and Chandel [4] in kiwifruit, and Ahmad et al. [5] in sweet cherry. The better growth of almond under fertigation might be due to continuous supply of nutrient during growth and development of almond as the fertilizers were applied in split doses. Increase in growth and yield of apple tree by fertigation was reported by Treder [18] and Reynolds et al. [19] in grapes.

The main trunk cross-sectional and area were primary, secondary and tertiary branches cross-sectional area influenced by nitrogen and potassium fertigation in almond (Table 2). Maximum cross-sectional area (72.67 cm^2^ and 90.28 cm^2^), primary branches (16.75 cm^2^ and 24.26 cm^2^), secondary branches (3.83 cm^2^ and 7.49 cm^2^), and tertiary branches (0.65 cm^2^ and 1.28 cm^2^) were recorded in T_2_ treatment and at par with T_4_ treatment and significantly superior to other treatments. The maximum TCSA of main trunk and primary, secondary, and tertiary branches showed better results because of better availability of the macronutrient as well as their effective utilization by the plants. Rao and Subramanyam [7] also observed that vegetative growth of pomegranate was positively related to the amount of nitrogen applied through drip and Ahmad et al. [5] in sweet cherry under karewa conditions of Kashmir valley.

### 3.2. Nut Characters

Nut number, weight, and yield were influenced by nitrogen and potassium fertigation (Table 3). Application of recommended dose of fertilizers through fertigation increases nut number in almond during 2011 and 2012. The highest nut number (990 and 3083 number/tree) was recorded in T_2_ treatment closely flowed by T_4_ treatment and significantly superior to recommended dose of fertilizers as basal application. Maximum nut weight and nut yield were recorded in T_4_ treatment which were enhanced by 16.66% and 7.18% in nut weight, 41.76% and 56.13% in nut yield per tree, and 41.26% and 46.90% in yield per hectare over T_1_ (RDF through soil application) during 2011 and 2012. Here, 25% fertilizers could be saved with fertigation practices. The minimum nut yield (1.74 and 3.06 kg/tree) was recorded in T_5_ treatment. The higher nut number with T_2_ treatment might be due to the fact that application of RDF through fertigation improves the nut retention in almond. The higher nut weight and yield in T_4_ treatment might be due to the fact that split application of 3/4 nitrogen and potassium as per requirement enhances the fertilizer-use efficiency. The higher fruit yield obtained under fertigation than soil fertilization may be explained in light of the hypothesis formulated by Bussi et al. [20], who suggest that fertigation results in higher yield due to direct effect of nutrient fertilizing timing and reduction in nutrient leaching that resulted in better fruit size and weight in peaches under fertigation compared with soil application. Ferrara et al. [21] reported increased yield level/vine by 25.6% with fertigation than the soil application. The smaller size of fruits in vines fertigated with 1/3 of recommended dose of NPK in the present study may be accounted for lower yield in this treatment. Dasberg and Erner [22] also observed gradual decline in mandarin fruit yield with fertigation at low nitrogen rate. Shirgure et al. [16] and Mahalakshmi et al. [23] observed an increase in fruit size and the weight increased with fertigation than soil application. Ahmad et al. [5] also reported similar observations in sweet cherry while working under Kashmir conditions.

**Figure 1:**
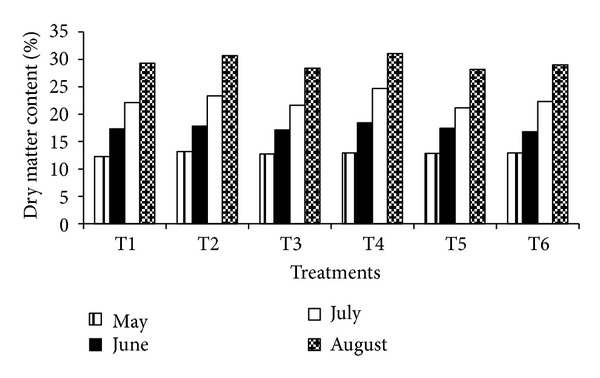
Nut dry matter accumulation under N&K fertigation in almond.

Nut dry weight at different stages of growth was influenced by nitrogen and potassium fertigation in almond (Figure 1). The nut dry weight increases with increasing the nut weight continue till maturity of nut from May to August under different fertigation treatment. Maximum nut dry matter content was recorded in T_4_ treatment in May and continued to be higher in subsequent nut growth and kernel filling stage. It was 5.21, 6.35, 11.71, and 6.08% higher over control treatment. This might be due to continuous supply of optimum nutrients as per phenological stages of nut growth and kernel filling in almond.

### 3.3. Leaf Nutrient Content

**Figure 2:**
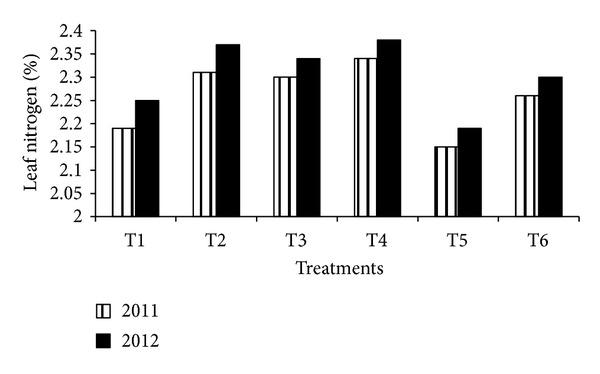
Leaf nitrogen content as influenced by N&K fertigation in almond.

**Figure 3:**
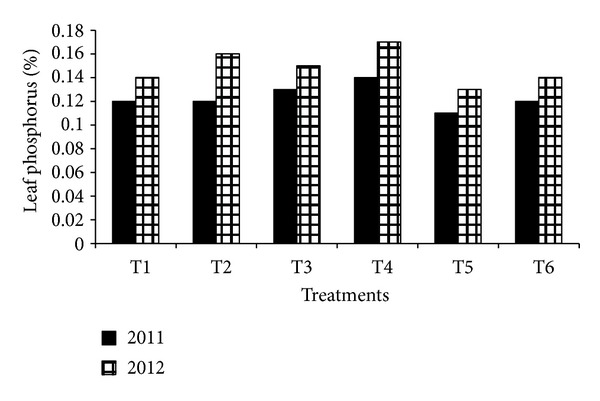
Leaf phosphorus as influenced by N&K fertigation in almond.

**Figure 4:**
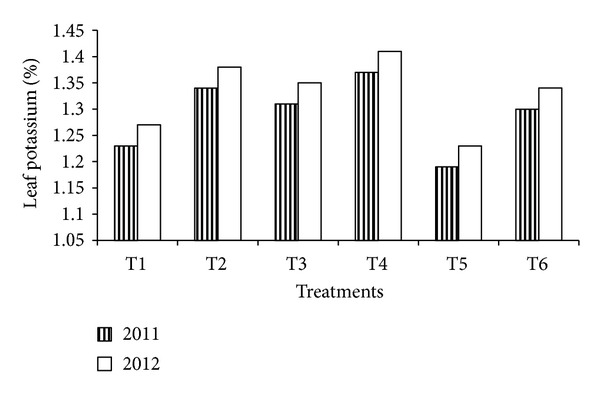
Leaf potassium as influenced by N&K fertigation in almond.

Leaf nutrient content as influenced by nitrogen and potassium fertigation in almond (Figures 2, 3, and 4). It is evident from the figure that almond tree fertigated with full and 3/4 of recommended dose of fertilizers had significantly higher leaf nutrient contents during both years. The highest leaf nitrogen (2.34 and 2.38%), phosphorus (0.14 and 0.17%), and potassium (1.37 and 1.41%) were estimated with T_4_ treatment closely followed by T_2_ treatment during 2011 and 2012. The increase availability of these elements under fertigation might have accounted for higher uptake of these nutrients. Similar increase in leaf nutrient content with application of higher dose of fertilizers has been reported by Murthy et al. [24] and Neilsen et al. [25].

This study suggests that 3/4 of recommended dose of fertilizer through fertigation (split application of N : K in the ratio of 2/3N : 1/3K at nut set to nut development and 1/3N : 2/3K at kernel filling to maturation stage) increases nut yield in almond by improving fertilizer-use efficiency and saving of 25% fertilizers as compared to 100% recommended dose of fertilizers under north western Himalayan region of India.

## Figures and Tables

**Table 1:** Vegetative growth as influenced by N&K fertigation in almond.

Treatments	Tree height (m)		Canopy volume (m^3^)		Shoot extension growth (cm)	
	2011	2012	2011	2012	2011	2012
T_1_	2.85	3.15	6.22	7.13	42.25	45.71
T_2_	2.98	3.23	7.15	8.11	49.46	52.04
T_3_	2.75	3.06	5.35	6.33	42.14	45.46
T_4_	3.21	3.56	6.85	7.77	47.12	50.46
T_5_	2.70	3.01	5.25	6.13	40.21	43.04
T_6_	2.89	3.20	5.29	6.26	41.75	43.12
LSD (*P* ≤ 0.05)	0.39	0.28	0.72	0.63	4.21	4.46

**Table 2:** Effect of N&K fertigation on primary, secondary, and tertiary branches in almond.

Treatments	Main trunk (cm^2^)		Primary branches (cm^2^)		Secondary branches (cm^2^)		Tertiary branches (cm^2^)	
	2011	2012	2011	2012	2011	2012	2011	2012
T_1_	52.95	77.18	15.96	23.31	3.63	6.87	0.42	1.09
T_2_	72.67	90.28	16.75	24.26	3.83	7.49	0.65	1.28
T_3_	60.91	82.02	14.85	21.31	3.23	6.55	0.42	1.11
T_4_	64.85	85.04	16.61	24.09	3.76	7.46	0.47	1.23
T_5_	49.76	73.93	10.86	16.53	2.06	5.14	0.40	0.86
T_6_	53.98	76.53	11.45	16.97	2.43	5.93	0.44	0.89
LSD (*P* ≤ 0.05)	12.12	8.23	2.24	3.13	0.75	1.05	0.12	0.21

**Table 3:** Effect of N&K fertigation on nut number and yield of almond.

Treatments	Nut number/tree		Nut wt. (g/nut)		Yield (kg/tree)		Yield (t/ha)	
	2011	2012	2011	2012	2011	2012	2011	2012
T_1_	727	2120	2.34	1.91	1.70	4.07	1.89	4.52
T_2_	990	3083	2.40	1.84	2.38	5.70	2.64	6.33
T_3_	729	2566	2.50	1.73	1.82	4.44	2.02	4.93
T_4_	882	3082	2.73	1.94	2.41	5.98	2.67	6.64
T_5_	675	1741	2.58	1.75	1.74	3.06	1.93	3.39
T_6_	735	2017	2.60	1.74	1.91	3.52	2.12	3.91
LSD (*P* ≤ 0.05)	152	450	0.12	0.10	0.32	1.12	0.35	0.97

## References

[1] Aslanta R, Guleryuz M, Tarun M, Ak BE (2001). Some chemical contents of selected almond (*Prunus amygdalus* Batsch) types. *11 GREMPA Seminar on Pistachios and Almonds*.

[2] Khan IA, Wani MS, Mir MA, Ahmed N, Mushtaq K, Hassan GI (2012). Response of almond (*Prunus dulcis*) to different drip irrigation levels vis-à-vis various phonological stages on flowering and yield of almond cv. Shalimar. *Indian Journal of Agricultural Sciences*.

[3] Reddy BMC, Srinivas K, Padma P, Raghupathi HB (2002). Response of *Robusta* banana to N and K fertigation. *Indian Journal of Horticulture*.

[4] Chauhan N, Chandel JS (2008). Effect of fertigation on growth, yield, fruit quality and fertilizer-use efficiency of kiwifruit (*Actinidia deliciosa*). *Indian Journal of Agricultural Sciences*.

[5] Ahmad MF, Samanta A, Jabeen A (2010). Response of sweet cherry (*Prunus avium*) to fertigation of nitrogen, phosphorus and potassium under Kerawa land of Kashmir valley. *Indian Journal of Agricultural Sciences*.

[6] Raina JN, Thakur BC, Suman S, Spehia RS (2005). Effect of fetrigation through drip irrigation system on nitrogen dynamics, growth, yield and quality of apricot. *Acta Horticulture*.

[7] Rao KD, Subramanyam K (2009). Effect of nitrogen fertigation on growth and yield of pomegranate var. Murdula under low rain fall zone. *Agriculture Science Digest*.

[8] Kumar D, Pandey V (2008). Effect of NPK fertigation on growth, yield and quality of banana “Rasthali” (AAB-Pathkapoora) in coastal agro-climatic conditions of eastern India. *Indian Journal of Agricultural Sciences*.

[9] Singh BP, Dimri DC, Singh SC (2007). Efficacy of NPK management through fertigation on growth characteristics of apple (*Malus domestica* Bork) plant. *Pantnagar Journal of Research*.

[10] Magen H (1995). Fertigation—an overview of some practical aspects. *Fertilizer News*.

[11] Rolston DE, Miller RJ, Schulbauch H, Nakayama FS, Bucks DA (1986). Management principles-fertilization. *Trickle Irrigation for Crop Production, Design, Operation and Management*.

[12] Wright H, Nichols D, Embree C (2006). Evaluating the accountability of trunk size and canopy volume models for determining apple tree production potential across diverse management regimes. *Acta Horticulturae*.

[13] Chapman HD (1964). Suggested foliar sampling and handling techniques for better determining the nutrient status of field and horticultural and plantation crops. *Indian Journal of Horticulture*.

[14] Jackon ML (1973). *Soil Chemical Analysis*.

[15] Steel RGT, Torrie JH (1986). *Principles and Procedures of Statistics*.

[16] Shirgure PS, Srivastava AK, Singh S (2001). Growth, yield and quality of Nagpur mandarin (*Citrus reticulata* Blanco) in relation to irrigation and fertigation. *Indian Journal of Agricultural Sciences*.

[17] Shirgure PS, Ram L, Marathe RA, Yadav RP (1999). Effect of nitrogen fertigation on vegetative growth and leaf nutrient content of acid lime (*Citrus aurantifolia*, Swingle) in Central India. *Indian Journal of Soil Conservation*.

[18] Treder W (2007). Influence of fertigation with nitrogen and a complete fertilizer on growth and yielding of “Gala” apple trees. *Journal of Fruit and Ornamental Plant Research*.

[19] Reynolds AG, Lowrey WD, de Savigny C (2005). Influence of irrigation and fertigation on fruit composition, vine performance, and water relations of Concord and Niagara grapevines. *The American Journal of Enology and Viticulture*.

[20] Bussi C, Huguet JG, Defrance H (1991). Fertilization scheduling in peach orchard under trickle irrigation. *Journal of Horticulture Science*.

[21] Ferrara E, Sorrenti M, Torricone A (2000). Fertigation of table grapes and assessment of quantity and quality. *Informatore Agrario*.

[22] Dasberg S, Erner Y (1997). The effects of irrigation management and nitrogen application on yield and quality of mincola mandarins. *Acta Horticulturae*.

[23] Mahalakshmi M, Kumar N, Jeyakumar P, Soorrianathasundaram K (2001). Fertigation studies in banana under normal system of planting. *South Indian Horticulture*.

[24] Murthy PV, Khan MM, Nachegowda V, Umamahes WP (2001). Effect of fertigation on leaf area and leaf petiole nutrient contents in Bangalore Blue grapes. *Current Research, University of Agricultural Sciences, Bangalore*.

[25] Neilsen GH, Neilsen D, Herbert LC, Hogue EJ (2004). Response of apple to fertigation of N and K under conditions susceptible to the development of K deficiency. *Journal of the American Society for Horticultural Science*.

